# The Challenges of Chagas Disease— Grim Outlook or Glimmer of Hope?

**DOI:** 10.1371/journal.pmed.0040332

**Published:** 2007-12-27

**Authors:** Rick L Tarleton, Richard Reithinger, Julio A Urbina, Uriel Kitron, Ricardo E Gürtler

## Abstract

The authors discuss the key challenges that undermine the control of Chagas disease and that must be urgently addressed to ensure long-term, sustainable control.

Through its impact on worker productivity, premature disability, and death, Chagas disease accounts for 670,000 disability-adjusted life years per annum [1]. This makes it the most important parasitic disease of the Americas. It is both a disease of poverty (Figures 1 and 2) and, like other neglected tropical diseases, also “poverty promoting” [2]. Traditionally confined to Latin America, Chagas disease is becoming an important health issue in the United States and Europe. First, due to the continuous influx of immigrants from disease-endemic countries in Latin America, a proportion of whom are infected with Trypanosoma cruzi, an increasing number of infected subjects are seen in clinical practice, whether, for example, through routine screening of US blood and organ banks [3] or physicians' offices in Europe [4]. The appearance of T. cruzi in US blood banks led to the implementation of the first Food and Drug Administration–approved diagnostic blood screening test for Chagas disease earlier this year [5]. Second, an increasing number of autochthonous Chagas disease cases have been reported in the US [6,7], which may mirror the increased reporting of T. cruzi infection in domestic animals and wildlife. Recognizing that Chagas disease can no longer be considered an “exotic” disease in the US, the American Society of Tropical Medicine and Hygiene held a clinical course in Chagas disease prior to its 2007 annual meeting (http://www.astmh.org/meetings/premeeting.cfm#clinical).

**Figure 1:**
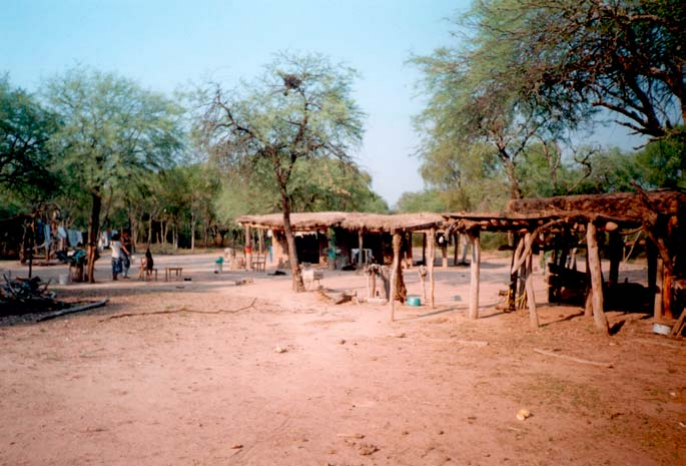
Chagas Disease Is Closely Linked to Poverty and Socioeconomic Development, and Poor Housing In Particular Example of a typical household environment in Chagas disease–endemic areas: mud-and-thatch house with a front veranda (in the background) and storage area (in the front), located in the dry Chaco forest around Amamá village, northern Argentina.

**Figure 2:**
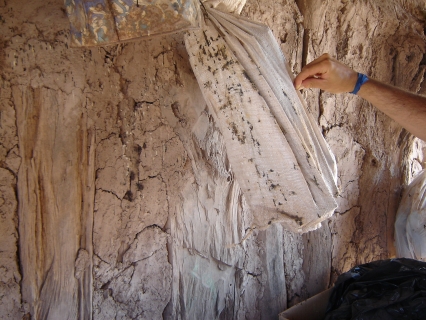
Interior of House Showing the Type of Wall Construction Often Found in Households in Many Endemic Areas Cracks in walls are commonly found, inhabited by insects, as evidenced by the insect feces on wall and wall hangings.

Despite estimates of up to 15 million existing cases and 50,000–200,000 new infections per year, funding for research, prevention, and control has been limited, and therapeutic options remain unsatisfactory. Consequently, several editorials and perspectives have recently drawn attention to Chagas disease and T. cruzi [8–10]. While these papers highlighted the impact of this disease on public health in the Americas, and rightly pointed out that major achievements have been made in its control, they failed to emphasize several key challenges that are currently undermining these achievements and that must be urgently addressed in order to move to the next stage: ensuring the long-term and sustainable control of this devastating disease.

## Etiology, Distribution, and Clinical Manifestations

Chagas disease (also known as American trypanosomiasis) is named for the Brazilian physician Carlos Chagas, who discovered the disease exactly a century ago and published its first description in 1909. It is caused by the protozoan parasite T. cruzi and is found in wildlife, domestic animals, and humans throughout much of rural as well as peri-urban areas of Mexico, Central America, and South America. In the US T. cruzi has been reported in dogs and a range of wild animals (e.g., raccoons and opossums), with human cases being relatively rare [6,7]. T. cruzi is mainly transmitted through blood-feeding triatomine bugs, but can also occur congenitally [7,11], through blood transfusion [12] or organ transplantation [13], and through the ingestion of contaminated food or fluids [14]. The complex life cycle involves different parasite life stages in both vector and host, all highly adapted to their respective environments, which maximizes transmission potential and/or host immune evasion and, hence, long-term parasite survival (http://www.dpd.cdc.gov/dpdx/HTML/TrypanosomiasisAmerican.htm#Life%20Cycle).

**Figure 3:**
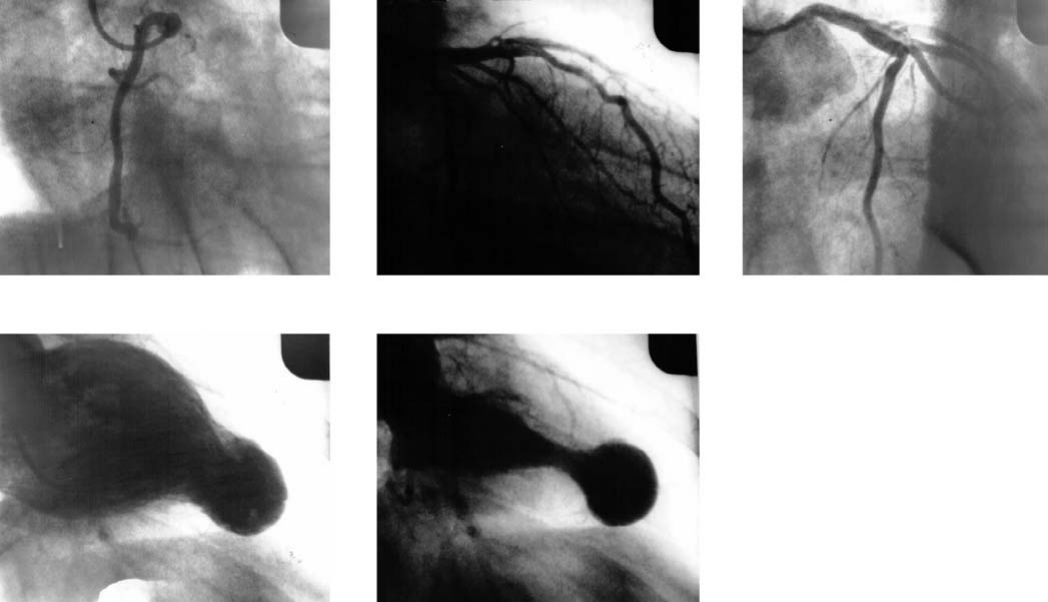
Cineventriculogram of a 49-Year-Old Male with Chagas Heart Disease The picture shows the apical aneurysm (sacular) with normal coronary arteries.

**Figure 4:**
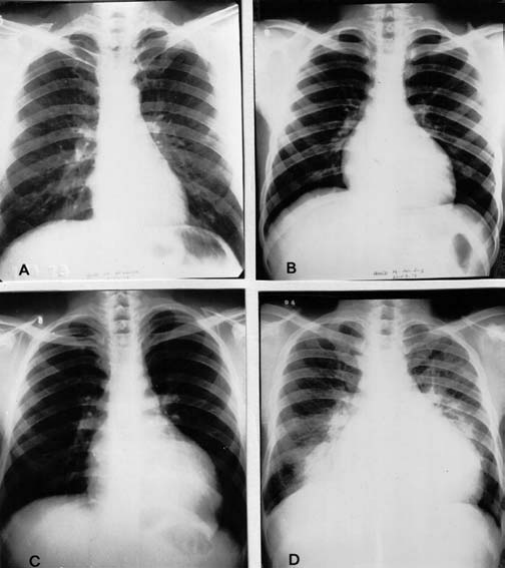
Chest X-Ray of 4 Different Patients with Chagas Heart Disease A: normal; B: mild cardiomegaly; C: moderate cardiomegaly; D: severe cardiomegaly with pulmonary congestion.

Most infected people do not know that they have become infected, as—in the disease's acute stage—the symptoms are benign (e.g., fever, swollen lymph glands, and, occasionally, an inflammatory reaction at the biting site or a swollen eye) or very rare (severe myocarditis or meningoencephalitis) [15]. Symptoms of acute infection may last up to a few weeks or months, and parasites may be found in the blood during this stage. Infections then remain largely asymptomatic (with few or no parasites found in the circulation), often for years or even decades, until up to 30% of patients develop chronic Chagas disease, i.e., cardiac or gastrointestinal complications, which if left untreated are severely debilitating and in many cases, fatal [15]. Chagas cardiomyopathy, which may result in cardiac arrhythmias, apical aneurysm, congestive heart failure, thromboembolism, and sudden cardiac death (Figures 3 and 4), is the most common form of cardiomyopathy in South and Central America and the leading cause of cardiovascular death in disease-endemic areas [16]. In people with suppressed immune systems (e.g., due to HIV/AIDS or chemotherapy), Chagas disease can reactivate with abundant parasites found in the blood and tissues. Because of its potentially long asymptomatic phase, however, Chagas disease is often considered a “silent killer” [9], which is one of several reasons why it fails to attract media attention.

## Grim Outlook 1: The Challenge of Vector Control

Tools to manage Chagas disease are numerous but are severely limited with regard to crucial aspects of prevention, detection, and treatment. Current vector control methods and strategies have significant limitations, diagnostics are variable and of unknown reliability, drugs for treatment are inadequate, and vaccines are nonexistent. Yet some bright spots exist, such as the decrease in transmission that has been achieved through control of insect vectors and screening of blood and blood products.

The “Southern Cone” initiative [17] has knocked down transmission rates dramatically in the southern tier of South America and is credited with interrupting vector-borne transmission via Triatoma infestans in Uruguay, Chile, and Brazil, primarily through insecticidal spraying of houses. However, elimination of T. infestans (one of the program's stated objectives) has not been achieved in the core of its distribution throughout the hyperendemic Gran Chaco region spanning northern Argentina, Paraguay, and Bolivia. Success may even be more difficult elsewhere in Latin America where there are many different vector species, each with distinct feeding and infestation behaviors, while in some endemic countries, notably Mexico, no control programs for this disease have ever been established. Among the many problems with exclusive reliance on insecticides is that prevention of re-infestation of houses by the insect vectors requires repeated spraying, which also promotes the development of insecticidal resistance—an outcome that is already documented in Argentina [18] and is increasingly detected in Bolivia. There are alternative insecticides, but their use is accompanied by added problems of higher costs or increased toxicity.

The decentralization of health services in many endemic countries combined with declining funding for control efforts has transferred the burden of control to provinces or local communities that are ill-equipped to supply these services. Even if vector control were better coordinated and fully funded, eradication of either the many domestic vector species or the parasites is not feasible for this zoonosis. Potentially, improvements in housing could permanently reduce vectorial transmission. However, at a minimum cost of US$200 to more than US$2,000 per house [19], such improvements will have to await significant economic development in endemic areas or specific investments for housing improvements on the order of US$10 billion.

## Grim Outlook 2: The Challenge of Chemotherapies and Vaccines

The current chemotherapies for T. cruzi infection and Chagas disease have many shortcomings, as do those in drug discovery and development. The available compounds for treatment, benznidazole (Rochagan, Roche Pharmaceuticals) and nifurtimox (Lampit, Bayer HealthCare), both have severe side effects, require long courses of treatment, and exhibit variable efficacy [20]. At present, the World Health Organization and other sources indicate that these drugs are active only in the acute and short-term (up to a few years) chronic phase (http://www.who.int/tdr/diseases/chagas/direction.htm). A number of studies now provide reason to question this recommendation [21–27]. Although some of these studies were non-controlled and used cure parameters that may be debatable because of the absence of a diagnostic gold standard (see below), they have been remarkably consistent in showing moderate to significant efficacy in long-term chronic infections. Thus, whilst waiting for the results of, for example, the BENEFIT trial (http://clinicaltrials.gov/ct/show/NCT00123916?order=1) evaluating the efficacy of benznidazole in chronic Chagas disease patients (expected to be completed in 2011), and unless or until better drugs are available, benznidazole and nifurtimox should be more widely used, based on the published evidence that such compounds may reduce the parasite burden and moderate disease progression in all stages of the disease [21–28]. It would therefore be ethically questionable to restrict the drugs' use to only patients with a defined duration of (acute and short-term chronic) infection, as currently recommended. However, the decision to treat chronic patients with these drugs should be made on a case-by-case basis, only after thorough clinical assessment and with continuous monitoring of potential side effects.

Academic and other noncommercial drug discovery efforts have yielded an increasing number of targets and new drug candidates, but surprisingly few of these promising leads have moved beyond the discovery/candidate stage. This stalemate is due in part to limited funding for further research and development, but even with drugs that have been available for more than 25 years, such as benznidazole, definitive preclinical evidence of cure in animal models is strikingly absent. A recent report from the Wellcome Trust–supported Pharmaceutical Research and Development Policy Project concluded that there is increasing industrial interest and progress in drug development for neglected diseases [29]—but this is certainly not the case for Chagas disease. Indeed, promising compounds such as Schering-Plough's posaconazole have yet to be further pursued as anti–T. cruzi drugs, despite extensive and promising preclinical data [20].

The success of drug treatment in arresting disease progression in subjects who have been infected for more than 20 years [21,22], coupled with a wealth of other data, has recently prompted a welcome shift toward studying Chagas disease as a problem of parasite persistence, rather than primarily as a problem of an inappropriate or imbalanced immune response [30]. This shift has also generated more interest in vaccines as a vehicle for control or treatment. Even so, vaccine development for T. cruzi infection has had a very slow start and remains nonexistent as a strategy for control or prevention. There are substantial potential problems with vaccines for Chagas disease, not the least of which include how one would test a conventional prophylactic vaccine for an infectious illness that is rarely detected until years or decades after the initial infection. However, given the cost-effective nature of vaccines and potential innovative applications (e.g., in transmission blocking or as therapeutics), more research is warranted, and vaccines should be included in the overall long-term strategy for control, prevention, and treatment of T. cruzi infection.

## Key Needs and Opportunities: Diagnostics and Integrated Vector Control

One of the key issues concerning Chagas disease is that of diagnosis. Without effective diagnostics, infected individuals cannot be identified and thus treated, and the effectiveness of treatment cannot be efficiently assessed. Moreover, the effectiveness of any control campaign, whether targeted at insect vectors, blocking of transmission, or vaccination of individuals, cannot be measured without competent diagnostics. Transmission via blood transfusion or tissue transplantation has been a point of concern for many years in Latin America but has only come to the fore in the US and Europe as the number of immigrants unknowingly carrying T. cruzi has increased. Most current serological tests, whether developed in-house or purchased commercially, employ crude antigen preparations from inappropriate parasite life-cycle stages (i.e., epimastigotes—which are present in the insect vector but not in mammalian hosts). Development of tests using one or more recombinant proteins/peptides may be an improvement, but even these tests often provide inconsistent and/or unreliable results [31–33]. The absence of a true gold standard (i.e., a method to consistently detect the presence of parasites in those individuals with T. cruzi infection) makes evaluation of the sensitivity of serological tests difficult. It is standard practice to use test sera that are positive on multiple other serological tests to assess the sensitivity of new tests, proving that any new test is no worse, but not necessarily any better, than existing ones. However, it is well documented that individuals with confirmed infection are typed as inconclusive or negative on multiple existing serological tests [34–36]. The design of tests to detect these inconclusive or “conventional seronegative” subjects has not been a priority. Likewise, the development of technologies that can more rapidly assess treatment efficacy, diagnose congenital infections, or determine the impact of transmission control methods has been slow. Fortunately, the development of sensitive, accurate, and practical diagnostic methods is a highly tractable problem, given an appropriate level of investment and interest. The development of highly sensitive and specific diagnostic field and laboratory tools to determine active infection is a crucial requirement for moving the entire field forward in the research, clinical, and public health arenas.

Although vector management has been the foundation of the overall strategy for prevention of T. cruzi infection, this area is also ripe for improvements. Despite the long-held promises of vector suppression through residual spraying with pyrethroid insecticides, current procedures often fail to eliminate triatomine bugs, especially in semiarid rural areas and in peridomestic habitats [37]. The lack of simple, sensitive tools for early detection of low-density populations of triatomine bugs that reappear after insecticide spraying undermines the effectiveness of control and elimination programs. Traditional high-risk settings require integrated control programs that are tailored to local environmental and sociocultural characteristics and employ a long-term perspective. The bust-and-boom cycles of the recent past, largely dependent on available funding, demonstrate that the effectiveness of Chagas disease vector control as currently practiced is limited.

On the other hand, progress in related research can help counter these shortcomings in vector control strategies. Our understanding of the eco-epidemiology of infection has significantly improved in recent years, especially with the application of geospatial analytical tools. These advances have allowed the development of infection and disease transmission models [38] as well as the more targeted planning of prevention and control strategies coupled with continued surveillance (e.g., identification of so-called transmission “hotspots” or loci of bug re-infestation) [39]. Several tools to prevent infection and to control (peri-) domestic bug populations have been evaluated, including insecticide-treated bed-nets [40] and dog collars [41], and optimum insecticide doses in peridomestic habitats. These tools have been shown to effectively reduce the contact of humans or reservoir dogs with the triatomine vector of T. cruzi. Bed-nets and collars would become particularly attractive options if they were used in an integrated approach aimed at controlling other vector-borne diseases such as malaria and leishmaniasis, respectively. The key issue is whether or not these new tools can be applied successfully at geographic scales ranging from individual villages to entire regions where current methods are not sufficient for the desired objective (control versus elimination).

## Glimmer of Hope

The problems of Chagas disease are many, but they are not insurmountable. There are numerous *partial* solutions already at hand that, if used in a coordinated manner, and with consideration of the unique characteristics of endemic areas (e.g., rural underdevelopment, poverty, lack of adequate housing, and increasingly decentralized health services), could have a significant impact. The entities that will fund and coordinate such an integrated effort remain to be identified, but clearly the involvement of the public sector is essential. Better diagnostics, drugs, and improved approaches to vector control programs will require more and better research, sharper focus, and greater rigor on the part of the research community. In addition, contributions from governments, the private sector, and nongovernmental organizations will be needed to establish the infrastructure for testing drugs and control methods as well as the platforms to develop and implement effective and affordable diagnostic tests. Fortunately, the research community has provided the majority of potential drug, vaccine, and diagnostic candidates and, via the sequencing of the T. cruzi genome and proteomes [42,43], has presented multiple leads for drug targets and diagnostic/vaccine candidates. However, neither for-profit nor nonprofit companies have taken on the challenges of developing these leads further. Effective scientific, philanthropic, and political leadership and forward-thinking coordination of a community effort in this realm, at both local and regional levels, is badly needed if we want to make significant inroads into this devastating disease. Ultimately, the success of such efforts will be heavily dependent on the long-term stability and prospects for economical, societal, and political development in the Americas.

## Supporting Information

Alternative Language Text S1 Translation of the article into Spanish by JAU (71 KB DOC).
