# Supplementary material for: The Challenges of Chagas Disease— Grim Outlook or Glimmer of Hope?
Source: PLoS Med. 2007 Dec 27;4(12):e332. doi: 10.1371/journal.pmed.0040332 (PMC2222930; doi:10.1371/journal.pmed.0040332)
Supplement: Alternative Language Text S1 — (71 KB DOC). [file pmed.0040332.sd001.doc]

**Los retos del Mal de Chagas – Sombrío Panorama o un Asomo de Esperanza?**

Rick L. Tarleton^1^, Richard Reithinger,^2,3,4^ Julio A. Urbina,^5,6^ Uriel Kitron,^7^ Ricardo E. Gürtler^8^

1 Center for Tropical and Emerging Global Diseases and Department of Cellular Biology, University of Georgia, Athens, GA, U.S.A.

2 Clinical Trials Area, Westat, Rockville, U.S.A.

3. School of Medicine and Health Sciences, George Washington University, Washington D.C., U.S.A.

4 London School of Hygiene and Tropical Medicine, London, U.K.

5 Instituto Venezolano de Investigaciones Científicas, Caracas, Venezuela.

6 Scientific Advisory Committee, Drugs for Neglected Diseases Initiative, Geneva, Switzerland.

7 Center for Zoonoses Research, University of Illinois, Champaign, U.S.A.

8 Department of Ecology, Genetics and Evolution, Universidad de Buenos Aires, Buenos Aires,

Argentina.

**Dirección de correspondencia:**

Rick L. Tarleton, Center for Tropical and Emerging Global Diseases, 500 D.W. Brooks Drive, University of Georgia, Athens, GA 30602 U.S.A. Email: Tarleton@uga.edu.

**Conflictos de Interés**

Los autores declaran no tener conflictos de interés con relación al contenido de este articulo.

**Introducción**

Por su impacto sobre la productividad en el trabajo, incapacitación prematura y muerte, la enfermedad de Chagas conlleva a la perdida de 670,000 años de vida ajustados por discapacidad [1]. Este hecho la hace la carga parasitaria humana mas importante de América Latina. Es una enfermedad asociada a la pobreza (Figuras 1 y 2) y, como muchas otras enfermedades no atendidas de las zonas tropicales, promotora de pobreza [2]. Aunque tradicionalmente era una enfermedad confinada a América Latina, la enfermedad de Chagas ha comenzado a manifestarse como ser un problema de salud pública en los EUA y Europa. Primero, porque debido al continuo influjo de inmigrantes a esos países provenientes de las zonas endémicas, entre los cuales ciertamente hay numerosos individuos infectados con *Trypanosoma cruzi*, se esta observando en la practica clínica un numero creciente de pacientes con esta infección, detectados por tamizajes de rutina de bancos de sangre u órganos en los EUA [3], en los consultorios médicos en Europa [4]. La frecuencia de los incidentes en los bancos de sangre ha llevado a la implementación a principios del 2007 de la primera prueba de diagnostico para la enfermedad de Chagas aprobada por la FDA (Federal Drug Administration o Agencia Federal para la regulación de Drogas) para su uso en los EUA [5]. Segundo, se ha notado tambien un incremento del numero de casos autóctonos de la dolencia en humanos en los EUA [6,7], que pueden estar asociados con el creciente numero de reportes sobre infecciones por *T. cruzi* en animales domésticos o silvestres. Reconociendo la urgencia de que esta enfermedad deje de ser considerada como una dolencia “exótica” en los EUA, la Sociedad Americana de Medicina Tropical e Higiene (ASTMH) esta organizando un curso clínico sobre enfermedad de Chagas en previo a su Reunión Anual del 2007 [http://www.astmh.org/meetings/premeeting.cfm#clinical].

A pesar de que se estima que el numero de personas actualmente infectadas puede alcanzar hasta 15 millones y de que ocurren 50.000 a 200.000 nuevas infecciones cada año, el apoyo financiero para investigación, prevención de la dolencia y control de su transmisión sigue siendo insatisfactorio. Como consecuencia, varios editoriales y perspectivas han sido publicados recientemente con el objetivo de llamar la atención sobre la enfermedad y su agente causal, el *T. cruzi*  [8-10]. Aunque tales comunicaciones hacen énfasis en el impacto del Mal de Chagas sobre la salud publica en el continente Americano y correctamente hacen notar los importantes avances que han ocurrido en el control de su transmisión, no mencionan varios serios retos que amenazan esos logros, los cuales tendrían que enfrentarse con urgencia para poder avanzar hacia la próxima etapa: garantizar el control sostenible y de largo plazo de esta devastadora dolencia.

**Etiología, Distribución y Manifestaciones clínicas**

La enfermedad o Mal de Chagas (tambien conocida como Tripanosomiasis Americana) debe su nombre al medico brasileño Carlos Chagas, quien la descubrió hace exactamente un siglo y publico su hallazgo en1909. La dolencia es causada por el protozoario parasito *T. cruzi* y esta ampliamente distribuida en animales salvajes y domésticos, así como en seres humanos en la mayor parte de las zonas rurales y peri-urbanas de México, Centro- y Sur América. En los EUA , el *T. cruzi* se ha reportado en perros y una variedad de mamíferos salvajes (como los mapaches y las zarigüeyas), en tanto que los casos humanos autóctonos son relativamente raros [6,7]. La transmisión del *T. cruzi* ocurre generalmente a través de insectos hematófagos (triatominos) pero tambien puede ocurrir por vía congénita [7,11], transfusiones de sangre [12], transplantes de órganos [13] o por la ingestión de comida y fluidos contaminados [14]. El ciclo de vida del parasito es complejo e incluye etapas tanto en el vector invertebrado como en el hospedero vertebrado, todas altamente adaptadas a sus ambientes respectivos, lo que maximiza el potencial de transmisión y/o la evasión del sistema inmune del hospedero y, como consecuencia, la sobrevida a largo plazo del parasito ([http://www.dpd.cdc.gov/dpdx/HTML/TrypanosomiasisAmerican.htm#Life%20Cycle](http://www.dpd.cdc.gov/dpdx/HTML/TrypanosomiasisAmerican.htm#Life%20Cycle)).

La mayoría de las personas infectadas no saben que han adquirido la infección, pues –en su fase aguda- los síntomas son raros o benignos (fiebre, inflamación de los ganglios linfáticos, ocasionalmente reacciones inflamatorias en el sitio de la picadura, edema ocular o, muy raramente, miocarditis severa o meningoencefalitis) [15]. Tales síntomas pueden durar algunas semanas o meses y los parásitos pueden ser encontrados en el torrente sanguíneo. La infección luego se mantiene generalmente asintomática (con parasitemia baja o indetectable) durante años e inclusive décadas, pero un 30% de los pacientes eventualmente desarrollan la forma crónica sintomática de la enfermedad, que incluye complicaciones cardiacas o gastrointestinales las cuales- si no son tratadas- pueden ser severamente debilitantes y en muchos casos fatales [15]. La miocardiopatía chagásica puede resultar en arritmias cardiacas, aneurisma apical, enfermedad cardiaca congestiva, trombo-embolismos y muerte súbita (Figuras 3 y 4), haciendo a esta enfermedad la causa mas común de cardiomiopatía en Centro- y Sur-América y la principal causa de muerte cardiovascular en las zonas endémicas del continente [16]. En personas con sistemas inmunológicos suprimidos (como consecuencia de VIH/SIDA o quimioterapia) la enfermedad de Chagas crónica se puede reactivar, con gran abundancia de parásitos en la sangre y otros tejidos del organismo. Debido al largo periodo de evolución de la infección asintomática, la enfermedad de Chagas es considerada un ‘asesino silencioso’ [9], siendo esta una de las varias razones por las cuales recibe poca atención de los parte de los medios de comunicación.

**Panorama Sombrío 1: El reto del control de vectores**

Hay numerosos instrumentos para el manejo de la enfermedad de Chagas. Sin embargo, los mismos tienen severas limitaciones en aspectos críticos de la prevención, detección y tratamiento de la dolencia: los métodos y estrategias de control de vectores tienen limitaciones importantes, el diagnostico es variable y de confiabilidad desconocida, las drogas para tratamiento específico son inadecuadas y no hay vacunas para prevenir esta infección. Aun así, hay puntos brillantes en el panorama, como lo son la marcada disminución en la transmisión de esta dolencia a través de programas de control de vectores y de las transfusiones sanguíneas. La Iniciativa del Cono Sur [17], ha reducido dramáticamente, principalmente por el rociado de insecticidas en las casas, las tasas de transmisión en los países de la zona sur de Sur-América y se la considera responsable de la interrupción de la transmisión de la dolencia a través del vector *Triatoma infestans* en Uruguay, Chile y Brasil. Sin embargo, la eliminación del *T. infestans* (uno de los objetivos del programa) no se ha alcanzado en el centro de distribución de esta especie, la zona hiperendémica del Gran Chaco, que incluye el norte de Argentina, así como Paraguay y Bolivia. El éxito será aun mas difícil en otras regiones de Latinoamérica, donde existen muy diferentes especies de vectores, cada una con diferentes hábitos de alimentación e infestación, en tanto que en algunos países endémicos –incluyendo el notorio caso de México – nunca han existido programas de control para esta enfermedad. Entre los muchos problemas asociados con la dependencia exclusiva en los insecticidas esta el hecho que la prevención de la reinfestación de las casas por los vectores requiere rociados repetitivos, lo cual promueve inexorablemente el desarrollo de resistencia al insecticida – un resultado que ya ha sido observado en Argentina [18] y que se esta detectando con cada vez mayor frecuencia en Bolivia. Existen insecticidas alternativos a los usados actualmente, pero su uso tiene como problemas asociados mayores costos y toxicidad. La descentralización de los servicios salud en muchos países endémicos, combinada con la reducción del apoyo económico a los programas de control, ha transferido el mayor peso del control de este tipo de dolencias a las provincias o autoridades locales, pobremente equipadas para prestar tales servicios. Aun si los programas de control de vectores estuvieran bien coordinados y dotados de los recursos necesarios, debe estar claro que la erradicación de muchos de los posibles vectores domésticos no es factible para esta zoonosis. Potencialmente, el mejoramiento de las viviendas podría llevar a una reducción permanente de la transmisión vectorial, pero a un costo estimado de 200 a > 2.000 $ EUA por casa [19], tal estrategia requeriría un avance significativo en el desarrollo socio-económico de las zonas endémicas del continente o un desembolso estimado actualmente en el orden de 10 millardos (10 mil millones) $ EUA.

**Panorama Sombrío 2: El reto de la quimioterapia y las vacunas**

Son numerosas las deficiencias de los recursos quimioterapéuticos para el tratamiento específico de las infecciones por *T. cruzi* y la enfermedad de Chagas, así como en el proceso de investigación y desarrollo de nuevas drogas. Las drogas actualmente disponibles, benznidazol (Rochagan®, Roche Pharmaceuticals) y nifurtimox (Lampit®, Bayer Healthcare), tienen ambas serios efectos colaterales, requieren largos periodos de tratamiento y su eficacia es muy variable [20]. Actualmente, la Organización Mundial de la Salud y otras fuentes hincan que esas drogas solo son activas en la fase aguda y la fase crónica temprana (pocos años de duración) de la enfermedad (http://www.who.int/tdr/diseases/chagas/direction.htm). Nuevos estudios parecen cuestionar esas recomendaciones [21-27]. Aunque algunos de los mismos no fueron controlados y utilizaron criterios de cura discutibles debido a la ausencia de un estándar oro (ver abajo), los mismos han demostrado que existe una eficacia moderada o significativa en infecciones de mayor duración. Así pues, mientras se completan estudios como el BENEFIT (<http://clinicaltrials.gov/ct/show/NCT00123916?order=1>), que evalúa la eficacia del benznidazol en pacientes con enfermedad de Chagas crónica (se espera que se complete el 2011) y hasta que nuevas y mejores drogas este disponibles, tanto benznidazol como nifurtimox deberían ser mas ampliamente usados pues hay evidencia publicada de que tales compuestos podrían reducir la carga parasitaria y moderar la progresión de la enfermedad in todas sus etapas [21-28]. Seria pues, éticamente cuestionable limitar su uso a pacientes con una particular duración de la dolencia (agua o crónica temprana), como se recomienda actualmente. Sin embargo, la decisión de tratar pacientes crónicos con tales drogas debe tomarse considerando la situación particular de cada individuo, luego de una evaluación clínica completa y con un monitoreo detallado de los posibles efectos colaterales.

La investigación académica y de otros grupos sin fines de lucro ha identificado un numero de blancos terapéuticos en este parasito pero, sorprendentemente, muy pocos estudios han continuado mas allá de la etapa de descubrimiento e identificación de candidatos a drogas. Tal estancamiento es debido, en parte, a los limitados fondos destinados a la investigación en esta área, pero aun en el caso de drogas que han estado disponibles por mas de 25 años, como el benznidazol, pruebas definitivas de cura en modelos animales están sorprendentemente ausentes de la literatura especializada. Un reporte de un reciente proyecto financiado por el Wellcome Trust sobre Políticas de Investigación y Desarrollo Farmacéutico concluye que hay un creciente interés del sector industrial y discernible progreso en el desarrollo de drogas para enfermedades no atendidas [29] – pero ciertamente ese no es el caso para la enfermedad de Chagas. En efecto, compuestos promisorios como el posaconazole (Schering-Plough) no han sido aun llevados a estudios clínicos como agente anti-*T. cruzi*, a pesar de numerosos y promisorios resultados preclínicos [20].

El éxito del tratamiento especifico en algunos pacientes con mas de 20 años de progresión de la enfermedad [21, 22], junto con un vasto conjuntos de otros datos experimentales, ha llevado a un bienvenido cambio de enfoque en el estudio de esta dolencia, de considerarla resultante de un sistema inmune inapropiado o no balanceado a estudiarla como el resultado de la prolongada persistencia del parasito [30]. Tal cambio ha generado tambien mas interés en el desarrollo de vacunas como un vehiculo para la prevención o tratamiento de esta parasitosis. Aun así, el desarrollo de una vacuna para infecciones por *T. cruzi* ha tenido un inicio muy lento y aun hoy la vacunación no juega ningún papel en el control de esta enfermedad. Hay serios problemas potenciales para el desarrollo de vacunas para la enfermedad de Chagas, entre ellos como probar el éxito de una vacuna profiláctica para una infección que generalmente se detecta años o décadas luego de la infección inicial. Sin embargo, el costo-efectividad de las vacunas, así como aplicaciones novedosas de las mismas (en el control de transmisión o en terapéutica) sugieren la importancia de hacer mas investigación en esta área y la inclusión de vacunas en la estrategia de largo plazo para el control, prevención y tratamiento de infecciones por *T. cruzi.*

**Necesidades y oportunidades claves: Diagnostico y control integrado de vectores**

Uno de los problemas claves con relación a la enfermedad de Chagas es el diagnostico – sin diagnostico efectivo no se pueden identificar y por ende tratar a los individuos infectados y la eficacia del tratamiento específico es difícil de evaluar. Mas aun, la efectividad de cualquier campaña de control, ya sea dirigida a vectores, bloqueo de la transmisión o vacunación de poblaciones no puede ser medida sin un competente sistema de diagnostico. La transmisión transfusional o por transplante de órganos ha sido un punto de preocupación por muchos años el Latinoamérica, pero solo ha llamado la atención en los EUA y Europa en tiempos recientes, cuando el numero de inmigrantes latinoamericanos, muchos de ellos portadores sin conocerlo del *T. cruzi,* se ha incrementado. La mayoría de los ensayos serológicos, ya sea desarrollados en las propias instituciones de los sistemas de salud o adquiridos comercialmente, emplean preparaciones crudas de antigenos totales de formas inapropiadas del ciclo de vida del parasito (generalmente la forma epimastigote, presente en el vector pero no en el hospedero vertebrado). Ensayos que utilizan una o varias proteínas o péptidos recombinantes pueden constituyen un avance, pero aun esos ensayos pueden dar resultados inconsistentes o no confiables [31-33]. La ausencia de un verdadero estándar oro (un método capaz de consistentemente detectar la presencia de parásitos en individuos con infección por *T. cruzi*) hace difícil la evaluación de la sensibilidad de los diferentes ensayos disponibles. La practica común es utilizar sueros de pacientes que han resultado positivos por varios otros ensayos serológicos, lo que demostraría que, de ser positivo el resultado, el nuevo método no es peor – pero tampoco mejor que las pruebas ya existentes. Sin embargo, esta bien documentado que individuos con infecciones demostradas pueden ser clasificados como “no conclusivos” o “dudosos” por varios de los métodos serológicos usados en la práctica clínica [34-36]. El diseño de métodos que permitan detectar esos pacientes infectados pero “no conclusivos” o negativos a la serología convencional no ha sido una prioridad hasta el momento. De igual manera, el desarrollo de tecnologías que permitan evaluar rápidamente la eficacia del tratamiento especifico, diagnosticar infecciones congénitas o determinar el impacto de programas de control de transmisión ha sido lento. Afortunadamente, hoy en día el desarrollo de métodos inmunológicos sensibles, precisos y prácticos es un problema manejable, si hay un nivel apropiado de interés e inversión. El desarrollo de un método de diagnostico sensible y especifico para la detección de infecciones activas, usable tanto en el campo como en el laboratorio, es un requerimiento crucial para avanzar en el estudio y manejo de la enfermedad, desde la investigación a la clínica y la salud pública.

Aunque el control de las poblaciones de vectores ha sido la base de la estrategia global para la prevención de infecciones causadas por *T. cruzi,* esta es tambien un área donde pueden hacerse muchas mejoras. A pesar de las viejas promesas de supresión de los vectores por el rociamiento con insecticidas piretroides de acción residual, los métodos actuales no son capaces de eliminar a los triatomíneos, particularmente en áreas semiáridas y en habitats peri-domésticos [37]. La ausencia de métodos simples y sensibles para detectar poblaciones de baja densidad que reaparecen después del rociamiento con insecticidas limita la efectividad de los programas de control y eliminación de esos insectos. Zonas tradicionalmente de alto riesgo requieren programas integrados de control diseñados específicamente para las características naturales y socio-económicas locales, con perspectivas de largo plazo. Los ciclos de éxito y fracaso observados en tiempos recientes en los programas de control, asociados a la disponibilidad de fondos, demuestran que la eficacia del modelo de control de vectores empleado actualmente es limitada. Por otro lado, el progreso en investigaciones asociadas a este campo de acción puede ayudar a superar sus limitaciones. Nuestra comprensión de la eco-epidemiología de la infección ha mejorado significativamente en los últimos años, especialmente con la aplicación de herramientas analíticas geo-espaciales. Esos avances han permitido el desarrollo de modelos de infección y transmisión de la enfermedad [38], así como una mas efectiva planificación de las estrategias de prevención y control, asociadas con vigilancia continua (identificación de las así llamadas “zonas calientes” o sitios de rápida reinfestación por los vectores) [39]. Varias herramientas para prevenir infestación y para el control de poblaciones peri-domésticas de vectores se han evaluado, incluyendo mosquiteros impregnados con insecticidas [40], collares para perros [41] y optimización de las dosis de insecticidas en los habitats peri-domésticos. Esas herramientas han demostrado ser capaces de reducir significativamente el contacto de los humanos o perros reservorios con las poblaciones de vectores del *T. cruzi*. Los mosquiteros impregnados con insecticidas y collares para perros podrían hacerse muy atractivos si se usan en programas integrados de control de vectores transmisores de otras infecciones, como malaria y leishmaniasis, respectivamente. La clave del asunto es si esas nuevas herramientas pueden o no aplicarse exitosamente en escalas geográficas que van desde villas individuales a regiones enteras, donde los métodos actuales son insuficientes para lograr el objetivo buscado (eliminación versus control).

**Un Asomo de Esperanza**

Los problemas que presenta la enfermedad de Chagas son múltiples, pero no son insuperables. Hay numerosas soluciones *parciales* ya disponibles que, si se usan en forma coordinada y tomando en consideración las características particulares de las áreas endémicas (sub-desarrollo rural, pobreza, ausencia de viviendas adecuadas y servicios de salud cada vez mas descentralizados), podrían tener un impacto significativo. Las entidades que deberían coordinar y apoyar económicamente ese esfuerzo coordinado aun no se han identificado, pero es evidente que la participación del sector publico es esencial. La introducción de mejores métodos de diagnostico, drogas y programas integrados de control de vectores requieren mas y mejor investigación, con mayor compromiso y rigurosidad de parte de la comunidad científica. Adicionalmente, las contribución de los gobiernos, el sector privado y Organizaciones No-Gubernamentales será necesaria para proveer la infraestructura donde puedan ensayarse las nuevas drogas y métodos de control, así como las plataformas para desarrollar e implementar pruebas diagnosticas efectivas y accesibles. Afortunadamente, la comunidad científica ha provisto la mayoría de los candidatos para drogas, vacunas y métodos de diagnostico y –por vía de la elucidación del genoma y proteoma del *T. cruzi* [42,43] – se ha identificado nuevas opciones para blancos terapéuticos y candidatos para diagnósticos o vacunas. Sin embargo, ninguna organización –con o sin fines de lucro- ha asumido el reto de hacer el ulterior desarrollo de tales hallazgos.

Un liderazgo efectivo en lo científico, filantrópico y político y la coordinación de los esfuerzos de las comunidades a nivel local y regional, con una visión de futuro, son esenciales para hacer avances contra esta devastadora dolencia. Sin embargo, el éxito de tales esfuerzos siempre dependerá de que se logre una estabilidad a largo plazo, así como avances en el desarrollo económico, social y político, en el continente americano.

**Agradecimientos**

Los autores agradecen a los Drs. Anis Rassi y Julio Lazzari por su observaciones criticas al manuscrito, a la Dra. Carla Cecere por la Figura 2 y al Dr. Rassi por las Figuras 3 y 4. REG proveyó la Figura 1. Las ideas discutidas en este documento se han beneficiado del apoyo a las actividades científicas de los autores por varias organizaciones de financiamiento a la investigación, incluyendo: U.S. National Institutes of Health (proyectos R01 AI-022070, R01 AI-033106 y P01 AI-044979 a RLT); The Burroughs Wellcome Fund (RLT), Sir Halley Stewart Trust (RR y REG); UNICEF/UNDP/World Bank/WHO Special Programme for Research and Training in Tropical Diseases (JAU); European Commission’s INCO-DEV Program (JAU); Howard Hughes Medical Institute (JAU); the Fogarty International Center and the National Institute of Environmental Health Sciences (U.S. National Institutes of Health / National Science Foundation Ecology of Infectious Disease Program, proyecto R01 TW05836 a UK y REG); Agencia Nacional de Promoción Científica y Técnica and the University of Buenos Aires (REG). Los autores no recibieron financiamiento para la elaboración del manuscrito. Las opiniones expresadas en este articulo son las de sus autores y no reflejan las de las instituciones las que se encuentran afiliados.

**Referencias**

1. World Health Organization (2004) The World Health Organization Report 2004. Changing History. Geneva: World Health Organization.

2. Hotez PJ, Molyneux DH, Fenwick A, Ottesen E, Ehrlich Sachs S, Sachs JD (2006) Incorporating a rapid-impact package for neglected tropical diseases with programs for HIV/AIDS, tuberculosis, and malaria. PLoS Med 3: e102.

3. Center for Disease Control and Prevention (2007) Blood donor screening for Chagas disease--United States, 2006-2007. MMWR Morb Mortal Wkly Rep. 56: 141-143.

4. Riera C, Guarro A, Kassab HE, Jorba JM, Castro M, et al. (2006) Congenital

transmission of *Trypanosoma cruzi* in Europe (Spain): a case report. Am J Trop Med Hyg. 75: 1078-1081.

5. Food and Drug Administration. Product approval information licensing action. ORTHO *T. cruzi* ELISA Test System. Available http://www.fda.gov/cber/products/tryorth121306.htm [Accessed May 23, 2007]

6. Beard CB, Pye G, Steurer FJ, Rodriguez R, Campman R, et al. (2003) Chagas disease in a domestic transmission cycle, southern Texas, USA. Emerg Infect Dis. 9:103-105.

7. Dorn P, Perniciaro L, Yabsley MJ, Roellig DM, Balsamo G, et al. (2007) Autochthonous Transmission of *Trypanosoma cruzi*, Louisiana. Emerg Infect Dis 13: 605-507.

8. Anonymous (2006) Chagas' disease--an epidemic that can no longer be ignored. Lancet 368: 619.

9. Maguire JH (2006) Chagas' disease--can we stop the deaths? N Engl J Med 355: 760-761.

10. Schofield CJ, Jannin J, Salvatella R (2006) The future of Chagas disease control. Trends Parasitol 22: 583-588.

11. Gurtler RE, Segura EL, Cohen JE (2003) Congenital transmission of *Trypanosoma cruzi* infection in Argentina. Emerg Infect Dis. 9: 29-32.

12. Young C, Losikoff P, Chawla A, Glasser L, Forman E (2007) Transfusion-acquired *Trypanosoma cruzi* infection. Transfusion 47, 540-547.

13. Center for Disease Control and Prevention (2006) Chagas disease after organ transplantation--Los Angeles, California, 2006. MMWR Morb Mortal Wkly Rep. 55: 798-800

14. Benchimol Barbosa PR (2006) The oral transmission of Chagas' disease: an acute form of infection responsible for regional outbreaks. Int J Cardiol. 112:132-133.

15. Prata A (2001) Clinical and epidemiological aspects of Chagas disease. Lancet Infect Dis 1: 92-100.

16. Rassi A Jr, Rassi A, Little WC, Xavier SS, Rassi SG, et al. (2006) Development and validation of a risk score for predicting death in Chagas' heart disease. N Engl J Med 355: 799-808.

17. Dias JC, Silveira AC, Schofield CJ (2002) The impact of Chagas disease control in Latin America: a review. Mem Inst Oswaldo Cruz 97: 603-612.

18. Picollo M, Vassena C, Santo Orihuela P, Barrios S, Zaidemberg M, et al. (2005) High resistance to pyrethroid insecticides associated with ineffective field treatments in *Triatoma infestans* (Hemiptera: Reduviidae) from Northern Argentina. J Med Entomol 42: 637-642.

19. Briceno-Leon R (1987) Rural housing for control of Chagas disease in Venezuela. Parasitol Today 3: 384-387.

20. Urbina JA, Docampo R (2003) Specific chemotherapy of Chagas disease: controversies and advances. Trends Parasitol 19: 495-501.

21. Viotti R, Vigliano C, Lococo B, Armenti H, LaPuente A, et al. (1998) Chronic chagasic cardiomyopathy: clinical and serologic evolution with and without benznidazole in long term follow-up. In: XIII World Congress of Cardiology; 1998; Brazil; 1998. p. 697-701.

22. Viotti R, Vigliano C, Lococo B, Bertocchi G, Petti M, et al. (2006) Long-term cardiac outcomes of treating chronic Chagas disease with benznidazole versus no treatment: a nonrandomized trial. Ann Intern Med 144: 724-734.

23. Andrade AL, Martelli CM, Oliveira RM, Silva SA, Aires AI, et al. (2004) Short report: benznidazole efficacy among *Trypanosoma cruzi*-infected adolescents after a six-year follow-up. Am J Trop Med Hyg 71: 594-597.

24. Pereira-Chioccola VL, Fragata-Filho AA, Levy AM, Rodrigues MM, Schenkman S (2003) Enzyme-linked immunoassay using recombinant trans-sialidase of *Trypanosoma cruz* can be employed for monitoring of patients with Chagas' disease after drug treatment. Clin Diagn Lab Immunol 10: 826-830.

25. de Andrade AL, Zicker F, de Oliveira RM, Almeida Silva S, Luquetti A, et al. (1996) Randomised trial of efficacy of benznidazole in treatment of early *Trypanosoma cruzi* infection. Lancet 348:1407-1413.

26. Sosa Estani S, Segura EL, Ruiz AM, Velazquez E, Porcel BM, Yampotis C (1998) Efficacy of chemotherapy with benznidazole in children in the indeterminate phase of Chagas' disease. Am J Trop Med Hyg 59: 526-529.

27. Sosa-Estani S, Segura EL (2006) Etiological treatment in patients infected by

*Trypanosoma cruzi*: experiences in Argentina. Curr Opin Infect Dis 19: 583-587.

28. de Castro AM, Luquetti AO, Rassi A, Chiari E, Galvao LM (2006) Detection of

parasitemia profiles by blood culture after treatment of chronic *Trypanosoma cruzi* infection. Parasitol. Res. 99, 379-383

29. Moran M, Ropars AL, Guzman J, Diaz J, Garrison C (2005) The new landscape of neglected disease drug development. London: The Wellcome Trust.

http://www.thegeorgeinstitute.org/shadomx/apps/fms/fmsdownload.cfm?file_uuid=F2B06396-EEA0-851E-3049-C9A030AEDE0F&siteName=iih. Accessed May 28, 2007.

30. Tarleton RL (2001) Parasite persistence in the aetiology of Chagas disease. Int J Parasitol 31: 549-553.

31. Pirard M, Iihoshi N, Boelaert M, Basanta P, Lopez F, Van der Stuyft P (2005) The validity of serologic tests for *Trypanosoma cruzi* and the effectiveness of transfusional screening strategies in a hyperendemic region. Transfusion 45: 554-561.

32. Caballero ZC, Sousa OE, Marques WP, Saez-Alquezar A, Umezawa ES (2007) Evaluation of serological tests to identify human *Trypanosoma cruzi* infection and cross reactivity with *Trypanosoma rangeli* and *Leishmania* spp cases. Clin Vaccine Immunol. 34, en prensa.

33. Silveira-Lacerda EP, Silva AG, Junior SF, Souza MA, Kesper N, et al. (2004) Chagas' disease: application of TESA-blot in inconclusive sera from a Brazilian blood bank. Vox Sang. 87:204-207.

34. Wincker P, Bosseno MF, Britto C, Yaksic N, Cardoso MA, et al. (1994) High correlation between Chagas' disease serology and PCR-based detection of *Trypanosoma cruzi* kinetoplast DNA in Bolivian children living in an endemic area. FEMS Microbiol Lett. 124: 419-423.
35. Salomone OA, Basquiera AL, Sembaj A, Aguerri AM, Reyes ME, et al. (2003)

*Trypanosoma cruzi* in persons without serologic evidence of disease, Argentina. Emerg Infect Dis. 9: 1558-1562.

36. Gutierrez R, Angulo VM, Tarazona Z, Britto C, Fernandes O (2004) Comparison of four serological tests for the diagnosis of Chagas disease in a Colombian endemic area. Parasitology. 129: 439-444.

37. Gurtler RE, Canale DM, Spillmann C, Stariolo R, Salomon OD, et al. (2004) Effectiveness of residual spraying with deltamethrin and permethrin on peridomestic populations of *Triatoma infestans* in rural western Argentina: a district-wide randomized trial. Bull WHO 82: 196-205.

38. Cohen JE, Gurtler RE (2001) Modeling household transmission of American

trypanosomiasis. Science 293: 694-698.

39. Cecere MC, Vazquez-Prokopec GM, Gurtler RE, Kitron U (2006) Reinfestation sources for Chagas disease vector, *Triatoma infestans*, Argentina. Emerg Infect Dis 12: 1096-1102.

40. Kroeger A, Villegas E, Ordonez-Gonzalez J, Pabon E, Scorza JV (2003) Prevention of the transmission of Chagas' disease with pyrethroid-impregnated materials. Am J Trop Med Hyg 68: 307-311.

41. Reithinger R, Ceballos L, Stariolo R, Davies CR, Gurtler RE (2006) Extinction of experimental *Triatoma infestans* populations following continuous exposure to dogs wearing deltamethrin-treated collars. Am J Trop Med Hyg 74: 766-771.

42. Atwood JA, 3rd, Weatherly DB, Minning TA, Bundy B, Cavola C, et al. (2005) The *Trypanosoma cruzi* proteome. Science 309: 473-476.

43. El-Sayed NM, Myler PJ, Bartholomeu DC, Nilsson D, Aggarwal G, et al. (2005) The genome sequence of *Trypanosoma cruzi*, etiologic agent of Chagas disease. Science 309: 409-415.

**Leyendas de Figuras:**

**Figura 1:**

La enfermedad o Mal de Chagas esta fuertemente asociada a la pobreza y limitado desarrollo socio-económico de las poblaciones humanas afectadas, en particular pobres condiciones habitacionales. Ejemplo de una vivienda familiar en una zona endémica para Mal de Chagas: una casa hecha de barro y hojas de palma, con una baranda a la entrada (fondo de la fotografía) y un deposito (frente de la fotografía), en una zona de bosque seco del Chaco, cerca del pueblo de Amamá, norte de Argentina.

**Figura 2:**

Interior de una casa, mostrando el tipo de paredes frecuentemente encontradas en las viviendas humanas de muchas zonas endémicas. Las grietas en las paredes son muy comunes y facilitan la colonización por los insectos vectores de Mal de Chagas, como se puede evidenciar por sus heces en las paredes y objetos colgados en las mismas.

**Figura 3:**

Cineventriculograma de un paciente masculino de 49 años, con miocardiopatía chagásica. La imagen muestra un típico aneurisma apical (sacular), con arterias coronarias normales.

**Figura 4:**

Imágenes radiológicas toráxicas de pacientes con Mal de Chagas. (A = Normal); (B = Cardiomegalia incipiente); (C = Cardiomegalia moderada); (D = Cardiomegalia severa, con congestión pulmonar).
